# Potential distribution and ecological impacts of *Acmella radicans* (Jacquin) R.K. Jansen (a new Yunnan invasive species record) in China

**DOI:** 10.1186/s12870-024-05191-5

**Published:** 2024-06-03

**Authors:** Shicai Shen, Fengping Zheng, Wei Zhang, Gaofeng Xu, Diyu Li, Shaosong Yang, Guimei Jin, David Roy Clements, Emma Nikkel, Aidong Chen, Yuchen Cui, Zewen Fan, Lun Yin, Fudou Zhang

**Affiliations:** 1 Key Laboratory of Prevention and Control of Biological Invasions, Ministry of Agriculture and Rural Affairs of China, Agricultural Environment and Resource Research Institute, Yunnan Academy of Agricultural Sciences, Kunming, China; 2 Key Laboratory of Green Prevention and Control of Agricultural Transboundary Pests of Yunnan Province, Agricultural Environment and Resource Research Institute, Yunnan Academy of Agricultural Sciences, Kunming, China; 3 Yunnan Lancang-Mekong Agricultural Bio-Security International Science and Technology Cooperation Joint Research Center, Kunming, China; 4 College of Ethnology and Sociology, Minzu University of China, Beijing, China; 5 Department of Biology, Trinity Western University, Langley, BC Canada; 6 Invasive Species Council of British Columbia, Williams Lake, BC Canada; 7 School of Agriculture, Yunnan University, Kunming, China; 8 School of Marxism, Southwest Forestry University and Southwest Research Center for Eco-civilization, National Forestry and Grassland Administration, Kunming, China

**Keywords:** New invasive species, *Acmella radicans*, Potential distribution, Predictive modeling, MaxEnt, Ecological impact

## Abstract

**Background Acmella radicans:**

(Jacquin) R.K. Jansen is a new invasive species record for Yunnan Province, China. Native to Central America, it has also been recently recorded invading other parts of Asia. To prevent this weed from becoming a serious issue, an assessment of its ecological impacts and potential distribution is needed. We predicted the potential distribution of *A. radicans* in China using the MaxEnt model and its ecological impacts on local plant communities and soil nutrients were explored.

**Results:**

Simulated training using model parameters produced an area under curve value of 0.974, providing a high degree of confidence in model predictions. Environmental variables with the greatest predictive power were precipitation of wettest month, isothermality, topsoil TEB (total exchangeable bases), and precipitation seasonality, with a cumulative contribution of more than 72.70% and a cumulative permutation importance of more than 69.20%. The predicted potential suitable area of *A. radicans* in China is concentrated in the southern region. Projected areas of *A. radicans* ranked as high and moderately suitable comprised 5425 and 26,338 km^2^, accounting for 0.06 and 0.27% of the Chinese mainland area, respectively. Over the 5 years of monitoring, the population density of *A. radicans* increased while at the same time the population density and importance values of most other plant species declined markedly. Community species richness, diversity, and evenness values significantly declined. Soil organic matter, total N, total P, available N, and available P concentrations decreased significantly with increasing plant cover of *A. radicans*, whereas pH, total K and available K increased.

**Conclusion:**

Our study was the first to show that *A. radicans* is predicted to expand its range in China and may profoundly affect plant communities, species diversity, and the soil environment. Early warning and monitoring of *A. radicans* must be pursued with greater vigilance in southern China to prevent its further spread.

## Background

Invasive alien species are defined as non-native species which have become naturalized through establishing self-sustaining populations in semi-natural or natural ecosystems [1]. Invasive species generally are species that may spread and colonize relatively large geographic areas [2]. The spread of invasive alien plant species has resulted in biodiversity loss, environmental problems, and great economic harm [3, 4]. In a review of biotic and economic impacts, Rai et al. [5] summarized the overall impacts as altering the composition but also structure and function, leading to ecological, economic, and social impacts. Invasive plants can reduce species richness and abundance of native species, alter soil chemistry and soil microbial communities, and disrupt community structure and ecosystem processes [6–8]. The effects of invasive plants on the biota are often apparent via comparisons with native plant species, over which they frequently have competitive advantages and fewer natural enemies present in the recipient environment [9, 10]. Rapid growth rates by comparison to native species frequently lead to the invasive plants dominating a given invaded environment at the expense of native communities [11]. The invasion process often includes four sequential stages: transport, introduction, establishment, and spread [12]. Early detection of invasive species new to an area, including assessing their potential impact, is the most cost-effective management strategy, proactively managing a species that has not yet spread into large areas of suitable habitat.

China, as the world’s third largest country, includes five climate zones: old-temperate, temperate, warm-temperate, subtropical, and tropical, which results in a rich biodiversity [13]. Almost any potential invasive alien species originating in one of these diverse climate zones throughout the world may find a suitable niche in China. Thus, with its varied topography, climatic conditions, and ecosystems, China is highly vulnerable to invasive alien species. Invasion by non-native species has accelerated via recent rapid increases in economic development, international trade, global tourism, and climate change in China [14, 15]. Many of the problematic invaders have been members of the Asteraceae family, such as *Mikania micrantha*, *Ageratina adenophora*, and *Eupatorium odoratum* [16].

Another more recent invasive plant invader in the Asteraceae family is *Acmella radicans* (Jacquin) R.K. Jansen. The plant is native to Central America but has recently been discovered in parts of Asia, including several new occurrence records in India [17–19]. In China, *A. radicans* was first found to be naturalized in Anhui Province in 2014 [20]. In 2017, *A. radicans* was first discovered as a potential issue in Yunnan Province during a survey of invasive alien plants. It was recorded and collected from Banhong Township, Cangyuan County. This plant was identified as *A. radicans* after a thorough survey of literature and expert opinion and the specimen was deposited in the Agricultural Environment and Resource Research Institute, Yunnan Academy of Agricultural Sciences, Kunming, China. Further field investigations and monitoring throughout the province showed *A. radicans* was widely distributed in Baoshan City and Lincang City as a serious invasive species, primarily invading farmland, tea gardens, orchard land, roadsides, and ditches. Even so, *A. radicans* is relatively early in its spread in the area, i.e., early in the fourth invasion stage, and thus is a good target for an “early detection, rapid response” approach. To carry out this approach, we need to understand its potential impacts and potential for further spread as examined in the current study. To date, literature predicting its ability to spread, and its potential ecological impacts is scarce, with the literature in Asia mostly limited to records of its occurrence without reference to impacts [17–19].

Species Distribution Models (SDMs) are valuable tools for studying the potential suitable distribution of invasive species [21] such as *A. radicans*. Commonly used SDMs include Maximum Entropy Model (MaxEnt) [22], Genetic Algorithm for Rule-set Prediction Model (GARP) [23], Biological Climatic Model (BIOCLIM) [24], and Match Climate and Compare Location Model (CLIMEX) [25]. Among these models, MaxEnt is the most widely used SDM at present. MaxEnt determines predicted species distributions through calculation of parameters with maximum entropy as a product of interactions between species and their environment [22]. MaxEnt and spatial analysis techniques have been used to predict the potential distribution of invasive plants such as *Xanthium italicum* [26], *Alternanthera philoxeroides* [27], *Erigeron canadensis* [28], *Lantana camara* [29], and many others. Many studies have confirmed the importance of MaxEnt model optimization, constrained by species distribution pattern, model conditions, validity of model assumptions and applicability of optimization measures, but model optimization may not necessarily improve the prediction ability [30–32]. Although MaxEnt model performs well in discrimination, due to many variables affecting species distribution, statistical models based on available distribution data often cannot reflect the complexity of the ecological demands of a given species [21, 33, 34]. As in any model system then, MaxEnt models provide a reasonable approximation of predicted distribution but are limited in the absence of detailed ecological information. Since SDMs focus on species distribution patterns, the relationship between predicted results and species ecological characteristics, such as population density, number of breeding populations, reproductive success, and other factors warrant further attention [34, 35]. Thus it is useful in studies such as ours to evaluate the model results in terms of the biology and ecology of the study species.

The objectives of the present study were to explore the potential distribution of *A. radicans* using a species distribution model (MaxEnt), examine ecological impacts of this new invasive plant species in China, and provide useful information to help minimize and mitigate invasion by *A. radicans*.

## Results

### Model optimization and evaluation of prediction precision

**Fig. 1:**
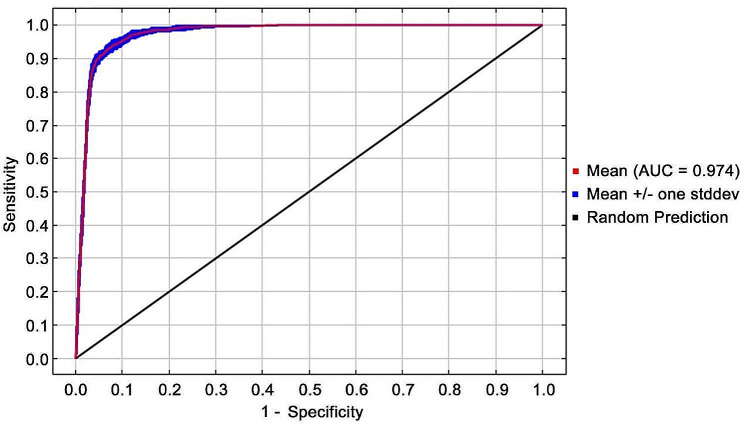
Receiver operating characteristic (ROC) test of the MaxEnt model (AUC is the area under curve)

Based on 396 *A. radicans* distribution records and 19 environmental predictor variables, the potential distribution in China of *A. radicans* was predicted using MaxEnt. With MaxEnt default parameter settings, RM = 1, FC = LQPTH, and delta AICc = 30.74. When RM = 1, FC = LPTH, delta AICc = 0, the model is optimal and omission rate at 5% is lower than the model under the default parameters (Table 1), which is 13.04% lower than the default value. Therefore, we set RM = 1 and FC = LPTH as the final parameters of the model. The AUC value of the simulated training under this parameter was 0.974 (Fig. 1), indicating a high level of confidence in model predictions.

**Table 1:** Evaluation results of MaxEnt model under different parameter settings

Setting	RM	FC	Delta AICc	Omission rate at 5%
Default	1	LQPTH	30.74	0.23232
Optimized	1	LPTH	0	0.20202

### Evaluation of environmental variables

**Fig. 2:**
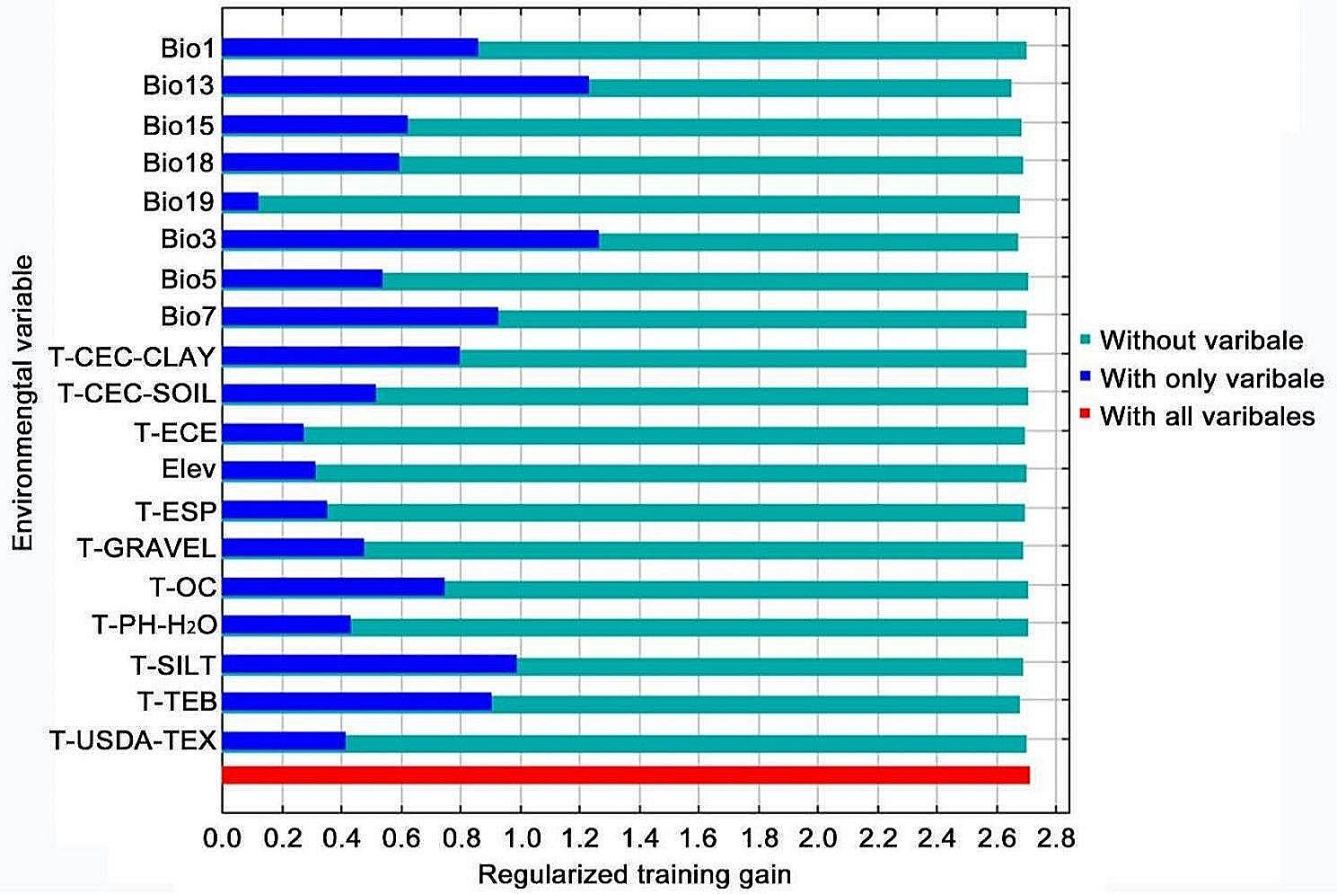
Jackknife test of the environmental variables

The Jackknife test was utilized to assess the degree of influence of environmental variables on the predicted outcome and thus determine how different variables contributed to the predicted potential distribution of *A. radicans*. The results of the Jackknife test (Fig. 2) showed that the top 8 environmental variables ranked from highest to lowest were isothermality (Bio3), precipitation of wettest month (Bio13), topsoil silt fraction (T-SILT), temperature annual range (Bio7), topsoil TEB (total exchangeable bases) (T-TEB), annual mean temperature (Bio1), topsoil1 CEC (T-CEC-CLAY), and topsoil organic carbon (T-OC) (i.e., ranked by regularization training gain when using only individual variables). Contribution rate and permutation importance value are also important indicators to assess the degree of influence of each environmental variable (Table 2). Precipitation of wettest month (Bio13), isothermality (Bio3), topsoil TEB (T-TEB), precipitation seasonality (Bio15), topsoil CEC (T-CEC-CLAY), precipitation of warmest quarter (Bio18), topsoil silt fraction (T-SILT), and annual mean temperature (Bio1), constituted the eight environmental variables contributing most to the potential suitability distribution of *A. radicans*, as evaluated via contribution rate and permutation importance values. The cumulative contribution rate reached 89.60% and the cumulative permutation importance value reached 82.10%. Combining the Jackknife test results, contribution rate, and permutation importance value analysis, we concluded that precipitation of wettest month (Bio13), isothermality (Bio3), topsoil TEB (T-TEB), and precipitation seasonality (Bio15) were the dominant variables affecting the potential suitability distribution of *A. radicans* (Table 2).

**Table 2:** Contribution rate and permutation importance value of each environmental variable (%)

Environmental variable	Contribution rate	Permutation importance value	Environmental variable	Contribution rate	Permutation importance value
Bio13	33.9	33.6	T-ECE	1.2	0.8
Bio3	17.9	10.7	Bio19	1.1	4.4
T-TEB	16.1	13.2	T-ESP	1.0	4.0
Bio15	4.8	11.7	Bio7	0.5	2.8
T-CEC-CLAY	4.6	0.3	T-CEC-SOIL	0.4	1.2
Bio18	4.3	4.3	T-OC	0.3	0.7
T-SILT	4.0	3.7	T-USDA-TEX	0.2	0.8
Bio1	4.0	4.6	T-PH-H_2_O	0.2	0.8
T-GRAVEL	3.3	1.4	Bio5	0.2	0.3
Elev	2.0	0.6			

### Potential distribution of *Acmella radicans*

**Fig. 3:**
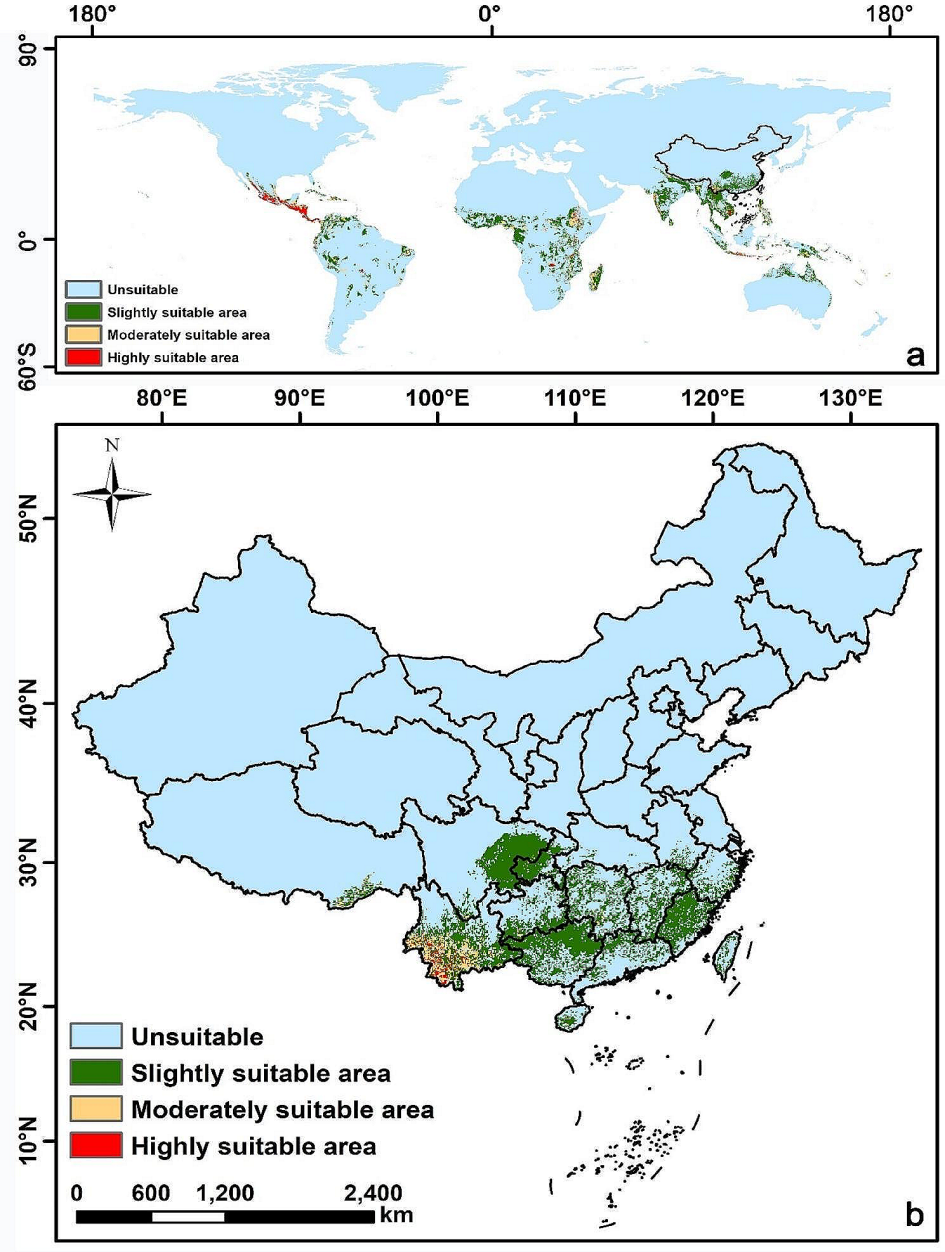
Potential suitable habitats of *Acmella radicans* predicted by the MaxEnt model. **a** Global potential geographical distribution (excluding Antarctica). **b** Potential geographical distribution in China

The potential suitable geographic area for *A. radicans* in China is mainly concentrated in the southern region (Fig. 3). The area characterized as slightly suitable covers 282,332 km², accounting for 2.94% of Chinese mainland. The slightly suitable area is chiefly distributed in eastern Sichuan, western Chongqing, southern Guizhou, northern Guangxi, all of Fujian, and a small amount in Hunan, Jiangxi, Zhejiang, and Guangdong.

The moderately suitable area of *A. radicans* in China covers 26,338 km², comprising 0.27% of the Chinese mainland. It is principally distributed in the western, southwestern, and south-central areas of Yunnan. Other potential distribution locales include southeastern Tibet, a small area encompassing southern Sichuan and northern Yunnan, the junction of southwestern Guizhou and northern Guangxi, south-central Hunan, central Hainan, and the eastern coastal region of Zhejiang.

The area deemed highly suitable for *A. radicans* in China covers 5425 km², accounting for 0.06% of the Chinese mainland. These highly suitable areas for *A. radicans* were concentrated in Yunnan and scattered in the west, southwest and south-central areas of Yunnan.

### Plant species and densities

The total area infested by *A. radicans* was over 5560 ha in Yunnan, primarily distributed in farmland, orchard land, roadsides, and wasteland in Baoshan City, and Lincang City. A total of 17 plant species belonging to 17 genera and 7 families were recorded within the study quadrats (Table 3). All 17 species were herbaceous and included 14 annual plants and 3 perennial plants, accounting for 82% and 18% of all species, respectively. Within communities of *A. radicans*, population densities of *A. radicans* were 19.81, 23.83, 40.41, 44.13, and 60.66 corresponding to study years 1, 2, 3, 4, and 5, respectively. Population density of most plant species declined markedly as the duration of the presence of *A. radicans* increased, however population density of *A. radicans* itself, increased significantly with increasing *A. radicans* growing period within five study years. Eight plant species *A. radicans*, *Ageratum conyzoides*, *Bidens pilosa*, *Borreria latifolia*, *Chloris virgata*, *Cynodon dactylon*, *Digitaria sanguinalis*, and *Setaria plicata* occurred over all five years and were dominant in *A. radicans* communities, having higher population densities than other species.

**Table 3:** Plant species and densities under different surveyed years from 2018 to 2022 (individual/m^2^) (mean ± SD)

Plant name	Life form	Plant density				
		1 year	2 years	3 years	4 years	5 years
*Acmella radicans*	AH	19.81 ± 0.34^e^	23.83 ± 0.47^d^	40.41 ± 1.67^c^	44.13 ± 1.17^b^	60.66 ± 1.09^a^
*Ageratum conyzoides*	AH	26.07 ± 0.62^a^	18.47 ± 0.41^b^	10.44 ± 0.14^c^	5.47 ± 0.34^d^	2.73 ± 0.10^e^
*Bidens pilosa*	AH	5.48 ± 0.13^a^	4.45 ± 0.14^b^	2.58 ± 0.08^c^	1.53 ± 0.04^d^	0.85 ± 0.06^e^
*Borreria latifolia*	AH	7.99 ± 0.19^a^	5.29 ± 0.15^b^	3.33 ± 0.19^c^	2.04 ± 0.11^d^	1.39 ± 0.14^e^
*Chenopodium album*	AH	1.10 ± 0.09^a^	0.78 ± 0.10^b^	0.30 ± 0.10^d^	0.51 ± 0.03^c^	0.21 ± 0.06^d^
*Chloris virgata*	AH	6.32 ± 0.16^a^	5.14 ± 0.25^b^	4.18 ± 0.20^c^	1.07 ± 0.08^d^	0.63 ± 0.15^e^
*Commelina benghalensis*	PH	1.64 ± 0.10^b^	1.92 ± 0.04^a^	1.68 ± 0.22^b^	0.85 ± 0.04^c^	0.59 ± 0.09^d^
*Conyza canadensis*	AH	1.47 ± 0.08^b^	1.72 ± 0.17^a^	1.27 ± 0.05^c^	0.64 ± 0.07^d^	0.36 ± 0.07^e^
*Crassocephalum crepidioides*	AH	0.66 ± 0.08^b^	0.83 ± 0.08^a^	0.47 ± 0.07^c^	0.29 ± 0.06^d^	0.14 ± 0.03^e^
*Cynodon dactylon*	AH	14.16 ± 0.21^a^	9.72 ± 0.32^b^	7.03 ± 0.16^c^	5.10 ± 0.18^d^	4.15 ± 0.18^e^
*Digitaria sanguinalis*	AH	27.29 ± 1.04^a^	20.63 ± 0.39^b^	10.77 ± 0.46^c^	5.78 ± 0.19^d^	3.32 ± 0.14^e^
*Echinochloa hispidula*	AH	3.43 ± 0.15^a^	3.13 ± 0.27^a^	1.66 ± 0.08^b^	0.53 ± 0.37^c^	0.00 ± 0.00^d^
*Eleusine indica*	AH	2.37 ± 0.15^a^	1.45 ± 0.09^b^	1.41 ± 0.07^b^	0.72 ± 0.12^c^	0.71 ± 0.11^c^
*Laggera pterodonta*	AH	0.97 ± 0.05^a^	0.98 ± 0.09^a^	0.54 ± 0.02^b^	0.43 ± 0.06^c^	0.13 ± 0.07^d^
*Setaria plicata*	PH	3.48 ± 0.20^a^	2.66 ± 0.11^b^	1.17 ± 0.10^c^	0.51 ± 0.05^d^	0.52 ± 0.02^d^
*Solanum indicum*	PH	0.20 ± 0.03^ab^	0.18 ± 0.13^ab^	0.13 ± 0.10^ab^	0.28 ± 0.19^a^	0.07 ± 0.09^b^
*Solanum nigrum*	AH	1.02 ± 0.10^a^	0.58 ± 0.04^b^	0.27 ± 0.03^c^	0.15 ± 0.04^d^	0.22 ± 0.04^cd^

Values with different letters in the same row are significantly different at the 0.05 level. AH = annual herb, PH = perennial herb

### Effects of *Acmella radicans* on plant importance values

There was variation in the response of individual species to increased duration of time over which *A. radicans* was present in the system. Importance values of *A. conyzoides*, *B. pilosa*, *B. latifolia*, *C. virgata*, *Commelina benghalensis*, *C. dactylon*, *D. sanguinalis*, *Echinochloa hispidula*, *Eleusine indica*, and *S. plicata* were higher within *A. radicans* communities, and declined markedly as the duration of the *A. radicans* infestation increased. Importance values of *A. radicans*, increased significantly the longer *A. radicans* was present. No trends were apparent for other species because of a high degree of variability across the *A. radicans* categories (Table 4).

**Table 4:** Plant importance values under different surveyed years (mean ± SD) (%)

Plant name	Life form	Importance value				
		1 year	2 years	3 years	4 years	5 years
*Acmella radicans*	AH	45.03 ± 0.52^e^	52.42 ± 1.99^d^	62.58 ± 1.83^c^	73.55 ± 1.11^b^	83.22 ± 0.89^a^
*Ageratum conyzoides*	AH	50.53 ± 2.49^a^	49.51 ± 1.47^a^	42.25 ± 1.40^b^	38.07 ± 1.10^c^	28.80 ± 1.01^d^
*Bidens pilosa*	AH	32.48 ± 2.08^a^	27.99 ± 0.85^b^	24.65 ± 0.69^c^	19.99 ± 0.93^d^	13.89 ± 0.94^e^
*Borreria latifolia*	AH	39.10 ± 0.81^a^	35.48 ± 0.53^b^	34.08 ± 0.69^b^	29.94 ± 1.10^c^	25.89 ± 1.48^d^
*Chenopodium album*	AH	14.50 ± 0.95^a^	11.65 ± 0.39^b^	6.91 ± 0.27^d^	9.19 ± 0.27^c^	4.24 ± 0.43^e^
*Chloris virgata*	AH	29.64 ± 1.48^a^	26.47 ± 0.58^b^	22.44 ± 1.10^c^	13.93 ± 0.79^d^	4.55 ± 0.55^e^
*Commelina benghalensis*	PH	19.11 ± 1.43^a^	19.80 ± 0.51^a^	19.55 ± 1.00^a^	13.85 ± 0.76^b^	10.09 ± 1.31^c^
*Conyza canadensis*	AH	14.86 ± 0.67^b^	20.38 ± 0.87^a^	13.87 ± 1.09^b^	11.18 ± 0.67^c^	8.04 ± 1.24^d^
*Crassocephalum crepidioides*	AH	13.40 ± 0.47^a^	13.50 ± 0.48^a^	9.45 ± 0.29^b^	7.09 ± 0.21^c^	2.37 ± 0.36^d^
*Cynodon dactylon*	AH	34.71 ± 0.97^a^	26.33 ± 0.51^b^	22.99 ± 0.72^c^	23.83 ± 0.45^c^	19.44 ± 1.02^d^
*Digitaria sanguinalis*	AH	45.07 ± 0.89^a^	42.98 ± 0.42^b^	40.34 ± 0.76^c^	32.43 ± 0.53^d^	29.70 ± 1.46^e^
*Echinochloa hispidula*	AH	21.37 ± 0.73^a^	17.48 ± 0.67^b^	14.58 ± 1.02^c^	5.59 ± 3.73^d^	0.00 ± 0.00^e^
*Eleusine indica*	AH	19.31 ± 0.64^a^	16.36 ± 0.43^b^	12.19 ± 0.26^c^	9.32 ± 0.31^d^	7.07 ± 0.15^e^
*Laggera pterodonta*	AH	13.42 ± 0.92^a^	12.35 ± 0.71^b^	9.81 ± 0.72^c^	7.29 ± 0.23^d^	2.16 ± 0.19^e^
*Setaria plicata*	PH	19.67 ± 1.15^a^	14.43 ± 0.64^b^	12.70 ± 0.96^c^	9.78 ± 0.74^d^	4.67 ± 0.18^e^
*Solanum indicum*	PH	4.38 ± 0.32^a^	2.04 ± 1.38^ab^	1.95 ± 1.30^ab^	3.67 ± 2.46^a^	1.17 ± 1.35^b^
*Solanum nigrum*	AH	11.79 ± 0.94^a^	9.24 ± 0.24^b^	6.93 ± 0.11^c^	4.62 ± 0.21^d^	4.65 ± 0.06^d^

Values with different letters in the same row are significantly different at the 0.05 level

### Effects of *Acmella radicans* on species diversity

The results demonstrated that the first and second years when *A. radicans* was present exhibited the highest species richness (17.00 and 16.75), Simpson index (0.864 and 0.854), Shannon-Wiener index (2.196 and 2.205), and Pielou index (0.773 and 0.775), followed by the subsequent three years of the *A. radicans* infestation (Table 5). Overall, species richness, diversity, and evenness parameters very rarely exhibited significant differences within the first two years. Over time, species richness, diversity, and evenness values significantly declined indicating that invasion of *A. radicans* resulted in decreased species richness, diversity indices, and evenness index of local plant species communities.

**Table 5:** Diversity indices of plant communities for different surveyed years (mean ± SD)

Years	Diversity indices			
	Species richness (S)	Simpson index (D)	Shannon-Wiener index (H)	Pielou index (J)
1	17.00 ± 0.00^a^	0.864 ± 0.004^a^	2.196 ± 0.005^a^	0.773 ± 0.003^a^
2	16.75 ± 0.50^a^	0.854 ± 0.004^b^	2.205 ± 0.005^a^	0.775 ± 0.002^a^
3	16.75 ± 0.50^a^	0.754 ± 0.007^c^	1.887 ± 0.005^b^	0.641 ± 0.004^b^
4	16.50 ± 0.58^a^	0.593 ± 0.003^d^	1.497 ± 0.010^c^	0.523 ± 0.003^c^
5	15.50 ± 0.58^b^	0.380 ± 0.001^e^	0.976 ± 0.005^d^	0.351 ± 0.002^d^

Values with different letters in the same column are significantly different at the 0.05 level

### Effects of ***Acmella radicans*** on soil characteristics

Among different *A. radicans* cover classes, soil characteristics varied significantly (Table 6). As the % cover of *A. radicans* increased, the pH, total K, and available K of the soil significantly increased; however, the organic matter, total N, total P, available N, and available P content of the soil declined significantly. It is evident that *A. radicans* may change the soil environment through absorbing more soil nutrients than other plants, which may facilitate its growth and invasion.

**Table 6:** Soil properties (i.e., pH, organic matter, total N, total P, total K, available N, available P, and available K) of different *Acmella radicans* % cover classes (mean ± SD)

Variables	Plant cover classes (%)				
	0	1–25	26–50	51–75	76–100
pH	5.17 ± 0.02^e^	5.24 ± 0.02^d^	5.33 ± 0.02^c^	5.47 ± 0.04^b^	5.62 ± 0.06^a^
Organic matter (g/kg)	37.90 ± 1.01^a^	36.33 ± 0.61^b^	31.27 ± 0.28^c^	29.42 ± 0.34^d^	25.59 ± 0.51^e^
Total N (g/kg)	1.70 ± 0.05^a^	1.55 ± 0.04^b^	1.48 ± 0.03^b^	1.24 ± 0.05^c^	1.08 ± 0.05^d^
Total P (g/kg)	1.22 ± 0.02^a^	1.11 ± 0.03^b^	1.02 ± 0.04^c^	0.94 ± 0.02^d^	0.93 ± 0.02^d^
Total K (g/kg)	12.77 ± 0.15^b^	13.00 ± 0.10^b^	13.13 ± 0.35^b^	13.13 ± 0.15^b^	14.73 ± 0.15^a^
Available N (mg/kg)	163.51 ± 6.50^a^	146.07 ± 3.09^b^	131.31 ± 3.43^c^	124.11 ± 2.27^c^	109.64 ± 5.01^d^
Available P (mg/kg)	78.43 ± 1.67^a^	61.25 ± 1.60^b^	49.24 ± 1.46^c^	37.62 ± 1.31^d^	33.53 ± 0.78^e^
Available K (mg/kg)	393.39 ± 8.89^e^	416.39 ± 7.29^d^	474.30 ± 8.81^c^	549.62 ± 5.23^b^	598.76 ± 17.45^a^

Different letters within the same row are significantly different at the 0.05 level

## Discussion

*Acmella radicans* is known to be a serious invasive plant in Bangladesh, Cuba, Curaçao, India, Tanzania, and Thailand [17–19, 36], but is rarely reported in China. We were the first to record the occurrence of *A. radicans* in Yunnan Province, China. Our field survey found that the total area infested by *A. radicans* was over 5560 ha in Yunnan, primarily distributed in farmland, orchard land, roadsides, and wasteland in Baoshan City and Lincang City. Our MaxEnt model revealed that the potentially suitable habitat in China for *A. radicans* was concentrated in southern China. The extent of areas rated as high and moderate suitability habitat areas for *A. radicans* in China were 5425 and 26,338 km^2^, accounting for 0.06 and 0.27% of the Chinese mainland area, respectively. The survival and expansion of invasive plant species are often favored by high rainfall and temperature levels in invaded regions [6, 8]. The study area is characterized by a subtropical climate with heavy rainfall and high temperature, which is suitable for plant growth of *A. radicans*. Moreover, the range of habitat suitability would likely increase under climate change.

The MaxEnt model approach has been extensively applied in species distribution simulation studies [37, 38] due to its advantages of being less sensitive to covariates in environmental variables [39], remaining stable with small sample sizes [40], and being able to fit complex variable relationships [41]. However, since the complex functional relationships in the MaxEnt model tend to lead to overfitting, the MaxEnt model is usually optimized by adjusting the parameters [42, 43]. Two important parameters that affect the MaxEnt model are the RM and FC. Currently, the ENMeval package [44], the SDMtune package [45], the Kuenm package [46], Wallace, Dismo and other open source software packages are effective in ascertaining the best parameter settings for the MaxEnt model. The Kuenm package allows MaxEnt to be used to establish a detailed and repeatable niche model, providing detailed model selection and calibration unavailable in other data package options [47]. In this study, we used the Kuenm package to find the optimal parameter settings of the MaxEnt model based on the mission rate at 5% and delta AICc. The fact the AUC value of the optimal model of 0.974 exceeded 0.9 indicated that the prediction performance of the MaxEnt model was improved by finding the optimal parameter settings, and that the possibility of over fitting the model was weakened.

It is important to evaluate potential spread of invasive plants based on local landscape factors [48]. Based on our observations in its present range in China, habitats *A. radicans* tends to invade include farmland, orchard land, roadsides, and wasteland. Throughout both moderately and highly suitable regions in its potential range in China, the landscape includes extensive areas of farmland producing similar crops to those found in its current range. Thus, the land area deemed moderately and highly suitable by our modelling is also vulnerable to the same kinds of impacts on plant community diversity and soil quality. Mountainous areas and other areas with extensive forest in the potential invaded range are likely the most serious barriers to spread; however, given the ability of *A. radicans* to grow on roadsides its ability is to invade these areas is highly probable as well. If *A. radicans* is likely to spread further in Yunnan and other parts of southern China as the SDM modelling predicts, it is also important to evaluate the seriousness of the impact on invaded plant communities. Invasive alien plants can reduce species richness, species diversity indices, evenness index, and native community structure and function [9, 49, 50]. The tendency of invasive plants to rapidly gain a competitive advantage over neighboring plants is attributed to their rapid growth, reproductive propagation ability, adaptation to a broad range of habitats, and strong allelopathic profiles [51–54]. By the second year of our five-year field survey, *A. radicans* had become the dominant and most densely populated species within the plant communities we monitored. Population density and importance values of many plant species, i.e., *(A) conyzoides*, *(B) pilosa*, *B. latifolia*, *(C) virgata*, *C. benghalensis*, *C. dactylon*, *(D) sanguinalis*, *(E) hispidula*, *E. indica*, and *S. plicata* declined significantly as *A. radicans* increased over time. Our field study found that the plant biomass, root number and depth, stem length, leaf area, and seed production number of *A. radicans* were markedly higher than those of neighboring plants, showing *A. radicans* achieved its competitive advantage via its extensive roots and larger aboveground size. Moreover, *A. radicans* may also have potential allelopathic compounds that could result in the inhibition of seed germination and seedling growth in neighboring plants [55]. Clearly, *A. radicans* is a strong competitor in this environment.

In addition to outcompeting neighboring plants, invasive alien plants can alter plant community structure and function in infested areas [9, 49]. Species richness and diversity of local plant communities was reduced 41% and 16% by *L. camara* invasion, respectively [56]. *Ageratina adenophora* caused a 68% reduction in species richness of understory vegetation in a *Pinus yunnanensis* forest, displacing countless native species in the process [57]. Similarly, the presence of *M. micrantha* was shown to decrease plant community parameters such as species richness and diversity [58]. Our results showed that the longer *A. radicans* was present in a plant community, the more species richness, diversity, and evenness values declined, likely threatening the stability of the system [59]. Thus, management of *A. radicans* is required in infested areas before it is too late.

Increasing numbers of studies have suggested that some invasive alien plants can effectively alter soil conditions, modifying soil chemistry, nutrient content, and nutrient availability, modifications which may actually lead to further invasion [60–62]. Soil nutrient concentrations have been observed to either increase or decrease in invaded habitats as compared with uninvaded habitats, depending on various factors [63–65]. For example, organic matter and concentrations of nitrogen, phosphorus, and potassium were lower in *Parthenium hysterophorus*-invaded soil than in non-invaded sites [64]. *Mikania micrantha* can likewise deplete soil nutrients, when growing either in monoculture or mixed culture with other plant species [50]. Our current study showed that the pH, total K, and available K in the soil increased significantly with increasing percent cover of *A. radicans*, but the organic matter, total N, total P, available N, and available P content of the soil declined significantly. Clearly *A. radicans* has the potential to change the soil environment through absorbing more soil nutrients, which may facilitate its growth and invasion.

## Conclusions

In conclusion, our results indicated that the MaxEnt model fit (AUC = 0.974) was robust and the most important environmental variables affecting the potential suitability of a habitat included precipitation of the wettest month, isothermality, topsoil TEB, and precipitation seasonality. Within China, potential suitable areas for *A. radicans* are mainly located in southern regions. Most suitable habitats for *A. radicans* are within Yunnan, whereas the moderately suitable habitats are distributed in parts of Yunnan as well as in some other provinces in southern China. In addition to evaluating the ability of *A. radicans* to expand its range, we looked at its potential impacts on invaded plant communities by evaluating species diversity within the current invaded range. Species richness, species diversity, and evenness of local communities were also reduced with increasing duration of *A. radicans* invasion. The importance values and population density of most plant species as well as species richness, diversity, and evenness values of local communities decreased with increasing duration of *A. radicans* infestation. Soil nutrients declined markedly with increasing *A. radicans* cover percentage. Hence, our study is a wakeup call for managers to be more aware of this species and develop increased monitoring and mitigation efforts before *A. radicans* becomes more widely distributed. Furthermore, given the relatively few studies on the biology and impacts of this species, we recommend pursuing more detailed research on the competitive mechanisms that result in the reduced plant community diversity observed in southern China. Because there is evidence that *A. radicans* produces phytochemicals harmful to other plants (Shen et al., unpublished), it would be particularly useful to research possible allelopathic mechanisms.

## Materials and methods

### Study site

**Fig. 4:**
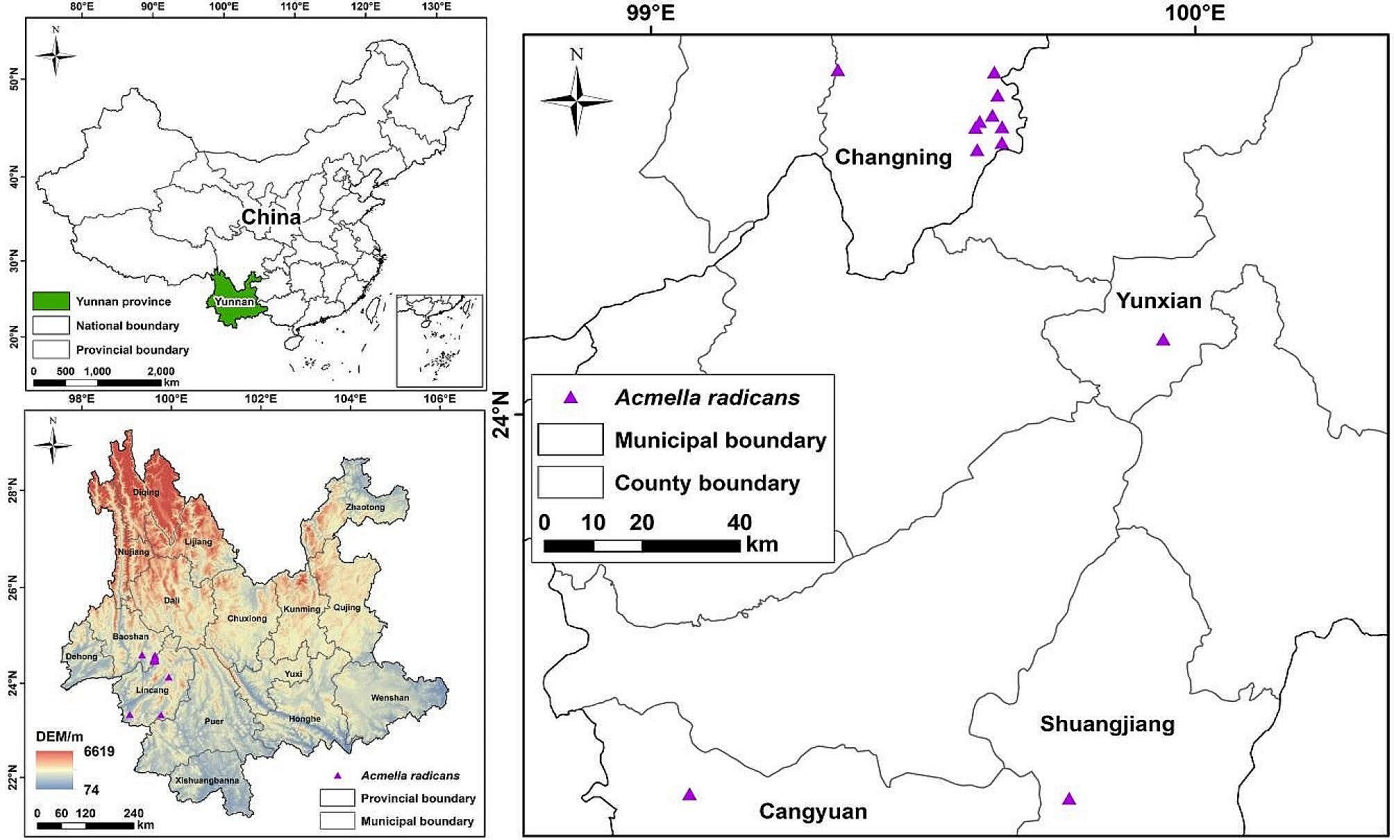
Distribution sampling site of *Acmella radicans* in Yunnan

Baoshan City (24°08′ N-25°51′ N, 98°05′ E-100°02′ E) and Lincang City (23°05′ N-25°03′ N, 98°40’-100°32’E) are neighboring and located in the west boundary area of Yunnan Province, Southwest China (Fig. 4). Baoshan shares a boundary of 167.78 km with Burma in the south and northwest, and Lincang shares a boundary of 290.79 km with Burma in the southwest [66, 67]. The study area is part of the “Gold Cross” for biological diversity of the earth [68, 69]. Baoshan and Lincang belong to the “Bridgehead-Golden Port” of Chinese southern Indian Ocean strategy and important “windows and gateways” of performance of reform and open policy along the frontier [70]. Two cities have a same subtropical low-latitude mountain plateau monsoon climate, characterized by warm winter, cool summer, abundant rainfall, and wet and dry seasons. The annual average temperature of Baoshan is 14–17 °C and the annual rainfall is 746.6–2095.2 mm. The annual average temperature and annual rainfall of Lincang are 16.5–19.6 °C and 1485.7 mm, respectively [66, 67]. Due to particular geographical and ecological conditions, Baoshan and Lincang are suitable for the growth of many tropical and subtropical invasive alien plants, including some new invasive species entering in Yunnan through long border with Burma.

### Study species

**Fig. 5:**
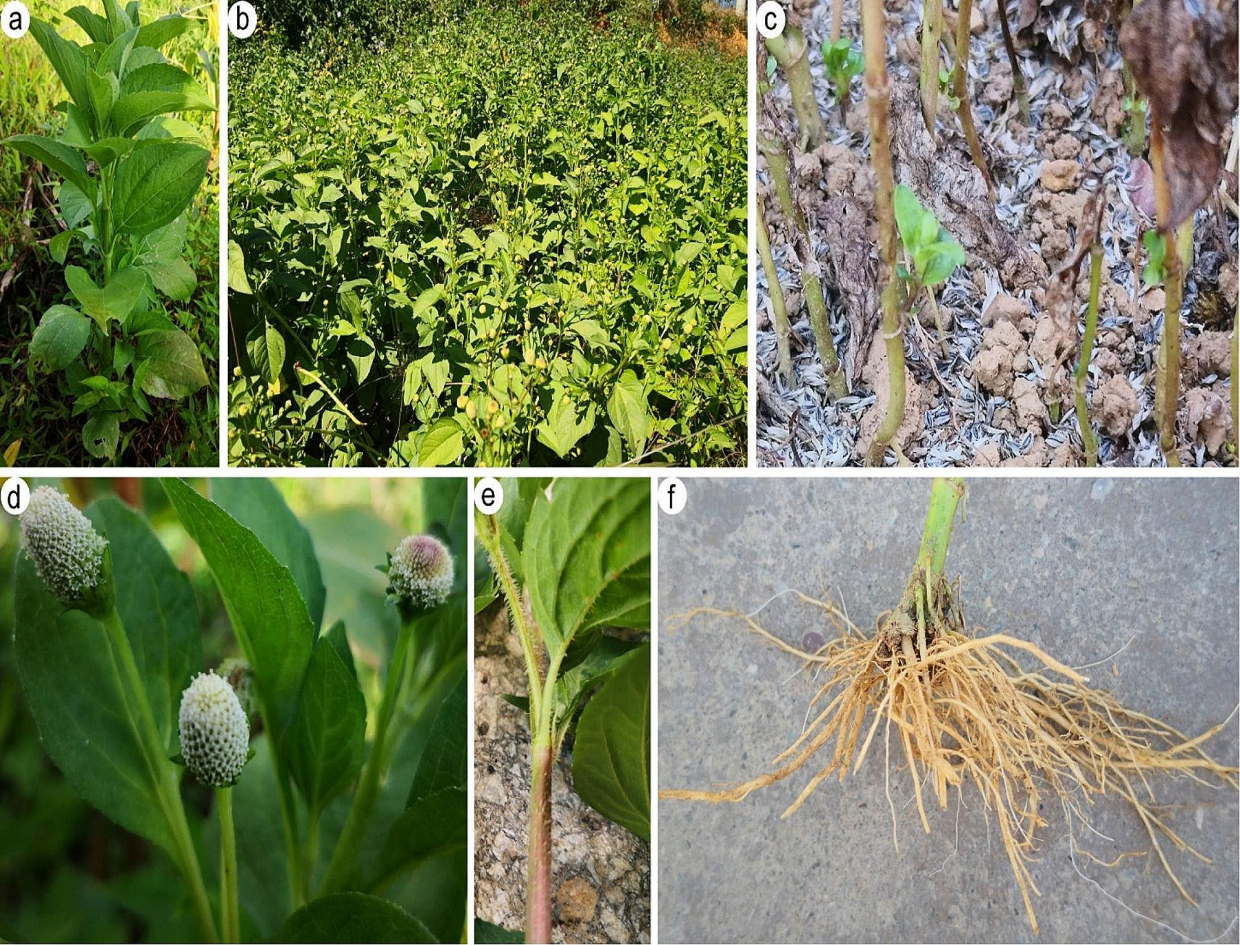
The plant habit (**a**, **b**), soil surface seed bank (**c**), inflorescence (**d**), stem (**e**), and root system (**f**) of *Acmella radicans*

*Acmella radicans*, is an annual erect herb in the Asteraceae family, native to Central America [17]. Some plant characteristic of *A. radicans* such as plant habit, soil surface seed bank, inflorescence, stem, and root system are shown in Fig. 5. Flowering and fruiting of *A. radicans* occurs between November and March and each plant can produce up to 14,300 seeds. This plant prefers moist habitats including riparian areas, ditches, and cultivated fields. Native to Central America introduced populations of *A. radicans* have been found in Bangladesh, Cuba, Curaçao, India, Tanzania, and Thailand [17–19, 36]. This plant is frequently used as a vegetable and in traditional medicine, particularly in its native range in meso-America [17, 55, 71]. Populations of *A. radicans* typically grow in riparian zones along streams producing many seeds, fostering continued range expansion through seed dispersal by water [20].

**Occurrence data of*Acmella radicans***.

In our field survey, 12 new distribution records were obtained in Yunnan Province, Southwest China (Fig. 4). Their specific distribution locations are listed in Table 7.

**Table 7:** Distribution locations of *Acmella radicans* in Yunnan

No.	City	County	Township	Village	Longitude	Latitude
1	Baoshan	Changning	Mengtong	Jiuyaozhai	99.64°E	24.55°N
2	Baoshan	Changning	Mengtong	Mengtong	99.63°E	24.52°N
3	Baoshan	Changning	Mengtong	Xinqing	99.64°E	24.50°N
4	Baoshan	Changning	Mengtong	Xiaomengtong	99.64°E	24.47°N
5	Baoshan	Changning	Mengtong	Changshan	99.60°E	24.46°N
6	Baoshan	Changning	Mengtong	Banjiazhai	99.60°E	24.50°N
7	Baoshan	Changning	Mengtong	Majiatian	99.60°E	24.50°N
8	Baoshan	Changning	Mengtong	Dahe	99.63°E	24.60°N
9	Baoshan	Changning	Wandian	Xiaojiezi	99.34°E	24.60°N
10	Lincang	Cangyuan	Banhong	Bamu	99.07°E	23.34°N
11	Lincang	Yunxian	Xingfu	Provincial Highway 313	99.94°E	24.13°N
12	Lincang	Shuangjiang	Bangbing	Menghuang Line	99.77°E	23.33°N

Data for 759 *A. radicans* distribution records were collected from the Global Biodiversity Information Facility (http://www.gbif.org/), the Chinese Virtual Herbarium (http://www.cvh.ac.cn/), and China National Knowledge Infrastructure (https://www.cnki.net/), including 5 distribution records from eastern China. The aforementioned data was combined with the 12 distribution records obtained from our field survey, and data considered invalid were removed. Additionally, to reduce model overfitting and improve model prediction precision, redundant data were removed using the function of removing duplicate occurrences (RDOc) in ENMtools [72] to ensure that only a single distribution point existed in the same grid, which resulted in 396 distribution records available for MaxEnt modeling.

### Bioclimatic and soil variables

The initial pool of environmental variables included 19 bioclimatic variables, 1 topographic variable (elevation), and 14 soil variables. The 14 soil variables comprised topsoil fractions for gravel, sand, silt, and clay as well as topsoil USDA texture classification, topsoil reference bulk density, topsoil organic carbon, topsoil pH (H_2_O), topsoi1 CEC (clay), topsoil CEC (soil), topsoil base saturation, topsoil TEB, topsoil sodicity (ESP), and topsoil salinity (Elco). Global bioclimatic variables and elevation data were from WorldClim (https://worldclim.org/) using Historical climate data (1970–2000), and global soil data from HWSD (https://www.fao.org/soils-portal/soil-survey/soil-maps-and-databases/harmonized-world-soil-database-v12/en/). All environmental variables were at a spatial resolution of 2.5 min. We utilized the world administrative map from the National Earth System Science Data Center, National Science and Technology Infrastructure of China.

**Fig. 6:**
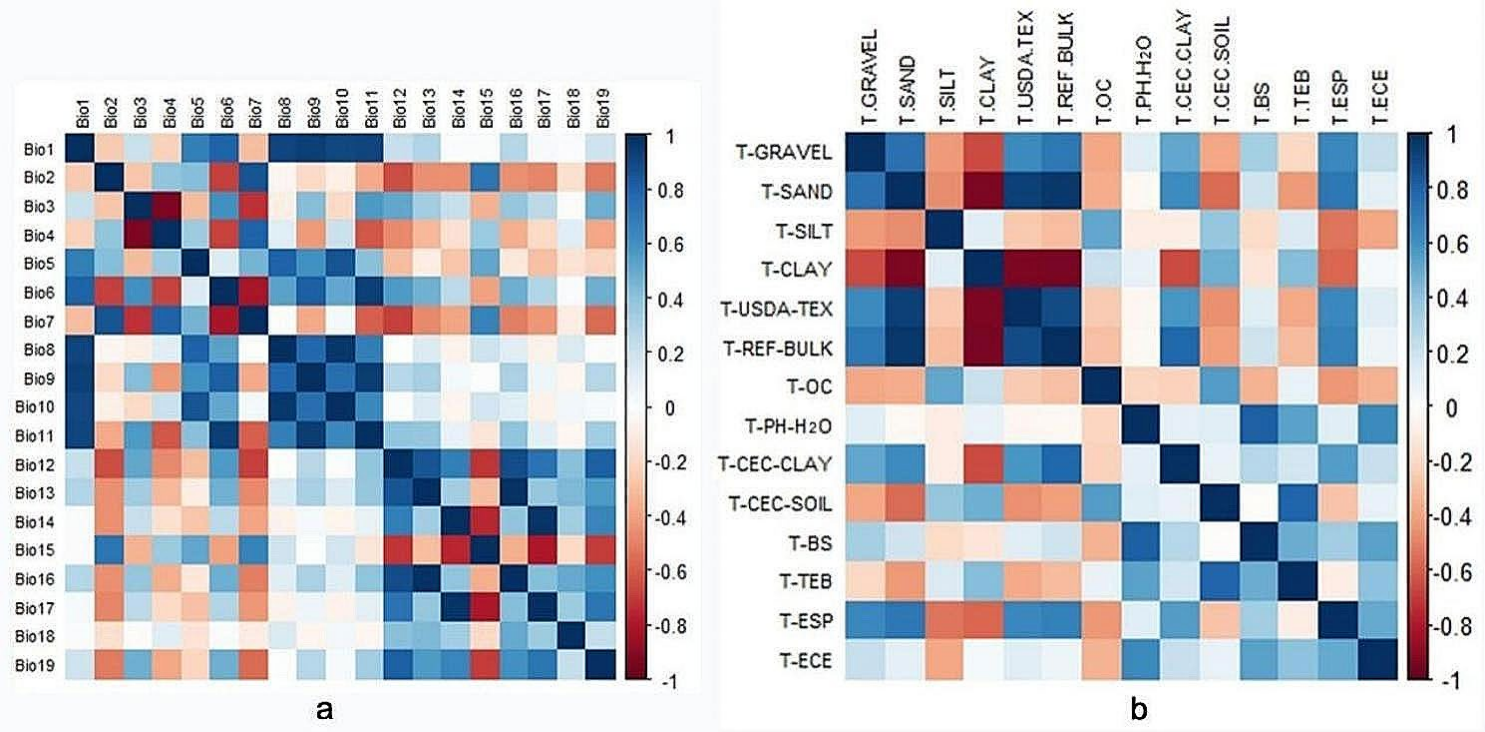
Pearson correlation coefficient. **a** Correlation coefficient matrix of 19 climate variables. **b** Correlation coefficient matrix of 14 soil variables

To avoid overfitting the model predictions due to covariance among environmental variables, correlation analysis of environmental variables was required prior to using them to construct our ecological niche model [73]. Therefore, we did Pearson correlation tests in SPSS 27 for the bioclimatic variables and soil variables (Fig. 6). The environmental variables with higher contribution rates were retained in combination with the Jackknife test results in the MaxEnt model, and 19 environmental variables from the initial pool were selected for MaxEnt modeling (Table 8).

**Table 8:** Variables used in the model prediction

Abbreviated name	Variable description
Bio1	Annual mean temperature
Bio3	Isothermality (BIO2/BIO7) (×100)
Bio5	Max temperature of warmest month
Bio7	Temperature annual range (BIO5-BIO6)
Bio13	Precipitation of wettest month
Bio15	Precipitation seasonality (coefficient of variation)
Bio18	Precipitation of warmest quarter
Bio19	Precipitation of coldest quarter
T-GRAVEL	Topsoil gravel content
T-SILT	Topsoil silt fraction
T-USDA-TEX	Topsoil USDA texture classification
T-OC	Topsoil organic carbon
T-PH-H_2_O	Topsoil pH (H_2_O)
T-CEC-CLAY	Topsoi1 CEC (clay)
T-CEC-SOIL	Topsoil CEC (soil)
T-TEB	Topsoil TEB
T-ESP	Topsoil sodicity (ESP)
T-ECE	Topsoil salinity (Elco)
Elev	Elevation

### MaxEnt model calibration, settings, and evaluation

The prediction performance of MaxEnt is influenced by two parameters: the regularization multiplier (RM) and feature combination (FC). We used the kuenm package in R (version 3.6.3) to adjust the parameters to achieve the detailed calibration, parameter selection, candidate model evaluation, and final model establishment of MaxEnt [46]. The RM value was set from 0.5 to 4.0, with each increment of 0.5, for a total of 8 selections. Additionally, 29 FCs of 5 features, including linear (L), quadratic (Q), product (P), threshold (T), and hinge (H), were selected for testing, including L, Q, P, T, H, LQ, LP, LT, LH, QP, QT, QH, PT, PH, TH, LQP, LQT, LQH, LPT, LPH, QPT, QPH, QTH, PTH, LQPT, LQPH, LQPH, LQTH, LPTH, LPTH, and LQPTH. Kuenm selected significant models from 232 candidate models with an omission rate of ≤ 5%, and then selected the model with delta AICc < 2 as the optimal model according to akaike information criterion (AICc) [46].

After parameterization according to the optimal model evaluated by kuenm, the species distribution point data and 19 environmental variables were imported by MaxEnt (version 3.4.4). A random sample of 75% of the distribution was selected as the training data set for modeling, and the remaining 25% of the distribution was used as the test data set to validate the model. The maximum number of background points was set to 10,000 and the maximum number of iterations to 500. The calculation results were then repeated 10 times using the Bootstrap method for the final average and we chose the Logistic output type. In the environment parameter setting, we evaluated the weight of each environmental variable using the Jackknife method and determined the dominant environment variable by combining the contribution rate and replacement important value of each environment variable. We used the area under curve (AUC) value of the receiver operating characteristic curve (ROC) to reflect the combined model sensitivity level and specificity [74]. An AUC value exceeding 0.8 indicates that the model predicts well, and an AUC value exceeding 0.9 indicates that the model predicts very well [75].

Finally, based on the global suitability threshold of *A. radicans* predicted by the MaxEnt model, and Janks natural breakpoint method was used to divide the suitability index of *A. radicans* into four classes: unsuitable (0 < *P* < 0. 05), slightly suitable area (0.05 < *P* < 0. 18), moderately suitable area (0.18 < *P* < 0. 41), and highly suitable area (*P* > 0.41), and obtained the potential distribution of *A. radicans* within China.

### Ecological impact assessment of *Acmella radicans*

To explore the ecological impacts of *A. radicans* on local plant communities and soil nutrient characteristics, thirty 1 m×1 m quadrats were randomly selected at each kind of habitats with four replicates in the same village in Changning County every year for five years since 2018. Climate and altitude did not differ among quadrats. Thus, a total of six hundred quadrats for five different habitats were surveyed each year for five years (2018 - year one up to 2022 – year five). Information collected from each quadrat consisted of plant species, plant density plant cover, frequency, and plant height for all species. Meanwhile, fifty soil samples were taken randomly from different *A. radicans* cover percentages (0%, 1–25%, 26–50%, 51–75%, and 76–100%) at the last field surveyed (2022) and then combined and treated as a single composite sample. The soil samples were then ground up, sifted through a 2 mm sieve, and air-dried at room temperature in preparation to analyze soil chemical characteristics. The pH, soil organic matter, total N, total P, total K, available N, available P, available K of soil nutrients were examined at the Soil Analysis and Detection Center of the Agricultural Environment and Resource Research Institute, Yunnan Academy of Agricultural Sciences, China.

Lastly, plant density, importance values and plant diversity parameters were calculated. The importance value (IV) for a given species was calculated as IV = (relative density + relative cover + relative frequency)/3 (Relative values were obtained via dividing species specific values by the sums of the densities, cover proportions and frequencies of all species in a plot, respectively) [52]. Simpson diversity index (D) [76] was calculated as D = 1-∑[N_i_(N_i_ − 1)/N(*N* − 1)], with N_i_ being the total number of individuals from i species, N the total number of individuals for all species in a plot. D values range from 0 to 1, from lowest to highest diversity. Shannon-Wiener diversity index (H) [77] was calculated as H = -∑P_i_lnP_i_, where P_i_ is the proportion of species i relative to the total number of species per plot. Pielou evenness index (J) [77] was calculated as J = H/lnS, where S is the species richness of each plot.

An analysis of variance (one-way ANOVA) was used to compare treatments. Duncan’s multiple range tests were used to compare treatments at a 5% significance level.

## Data Availability

The raw data supporting the conclusions of this article will be made available by the corresponding author Fudou Zhang, without reservation.
